# Supplementary material for: Pharmacist-Initiated Pre-Emptive Pharmacogenetic Panel Testing with Clinical Decision Support in Primary Care: Record of PGx Results and Real-World Impact
Source: Genes (Basel). 2019 May 29;10(6):416. doi: 10.3390/genes10060416 (PMC6628264; doi:10.3390/genes10060416)
Supplement: Supplementary file 1 [file genes-10-00416-s001.pdf]

**Supplementary Table S1.** The tested PGx Panel in the IP3 pilot study.

| Gene | Allele | Reference Sequence + Variant | RS-number |
|---|---|---|---|
| CYP2C9 | *2 | NG_008385.1:g.3608C>T | rs1799853 |
| CYP2C9 | *3 | NG_008385.1:g.42614A>C | rs1057910 |
| CYP2C19 | *2 | NG_008384.3:g.19154G>A | rs4244285 |
| CYP2C19 | *3 | NG_008384.3:g.17948G>A | rs4986893 |
| CYP2C19 | *17 | NG_008384.3:g.-806C>T | rs12248560 |
| CYP2D6 | *2A | M33388:g.-1584C>G | rs1080985 |
| CYP2D6 | *10 | M33388:g.100C>T | rs1065852 |
| CYP2D6 | *12 | M33388:g.124G>A | rs5030862 |
| CYP2D6 | *11 | M33388:g.883G>C | rs201377835 |
| CYP2D6 | *17 | M33388:g.1023C>T | rs28371706 |
| CYP2D6 | | M33388:g.1661G>C | rs1058164 |
| CYP2D6 | *6 | M33388:g.1707delT | rs5030655 |
| CYP2D6 | *4 | M33388:g.1846G>A | rs3892097 |
| CYP2D6 | *40 | M33388:g.1863_1864insTTTCGCCCCTTTCGCCCC | rs72549356 |
| CYP2D6 | *20 | M33388:g.1973_1974insG | rs72549354 |
| CYP2D6 | *19 | M33388:g.2539delAACT | rs72549353 |
| CYP2D6 | *3 | M33388:g.2549delA | rs35742686 |
| CYP2D6 | *38 | M33388:g.2587delGACT | rs72549351 |
| CYP2D6 | *9 | M33388:g.2615delAAG | rs5030656 |
| CYP2D6 | | M33388:g.2850C>T | rs16947 |
| CYP2D6 | *7 | M33388:g.2935A>C | rs5030867 |
| CYP2D6 | *44 | M33388:g.2950G>C | rs72549349 |
| CYP2D6 | *41 | M33388:g.2988G>A | rs28371725 |
| CYP2D6 | *29 | M33388:g.3183G>A | rs59421388 |
| CYP2D6 | *42 | M33388:g.3259_3260insGT | rs72549346 |
| CYP2D6 | *18 | M33388:g.4132_4133insGTGCCCACT | rs1135836 |
| CYP2D6 | | M33388:g.4180G>C | rs1135840 |
| CYP2D6 | *5 | NC_000022.10:g. | |
| CYP2D6 | xN | duplication | |
| CYP3A5 | *3 | NG_007938.1:g.12083G>A | rs776746 |
| CYP3A5 | *6 | NG_007938.1:g.19787G>A | rs10264272 |
| DPYD | *2A | NM_000110.3:c.1905+1G>A | rs3918290 |
| DPYD | *13 | NM_000110.3:c.1679T>G | rs55886062 |
| DPYD | | NM_000110.3:c.1236G>A | rs56038477 |
| DPYD | | NM_000110.3:c.2846A>T | rs67376798 |
| SLCO1B1 | | NM_006446.4:c.521T>C | rs4149056 |
| TPMT | *2 | NM_000367.4:c.238G>C | rs1800462 |
| TPMT | *3B | NM_000367.4:c.460G>A | rs1800460 |
| TPMT | *3C | NM_000367.4:c.719A>G | rs1142345 |
| VKORC1 | | NM_206824.2:c.173+1000C>T | rs9934438 |

**Supplementary Table S2.** Actionable drug-gene interactions relevant to the panel used (*n* = 41).

| Gene | Interacting drugs for which actionable DPWG guidelines are available | Actionable phenotypes |
|---|---|---|
| *CYP2C9* | phenytoin | PM, IM, *1/*2, *1/*3, *2/*2, *2/*3, *3/*3 |
| *CYP2C19* | **citalopram** | PM, IM |
| | **clopidogrel** | PM, IM |
| | **escitalopram** | PM, IM, UM |
| | **imipramine** | PM |
| | **lansoprazole** | UM |
| | **omeprazole** | UM |
| | **pantoprazole** | UM |
| | **sertraline** | PM |
| | **voriconazole** | PM, IM, UM |
| *CYP2D6* | **amitriptyline** | PM, IM, UM |
| | **aripiprazole** | PM |
| | **atomoxetine** | PM, IM, UM |
| | **clomipramine** | PM, IM, UM |
| | **codeine** | PM, IM, UM |
| | **doxepin** | PM, IM, UM |
| | eliglustat | PM, IM, UM |
| | **flecainide** | PM, IM, UM |
| | **haloperidol** | PM, UM |
| | **imipramine** | PM, IM, UM |
| | **metoprolol** | PM, IM, UM |
| | **nortriptyline** | PM, IM, UM |
| | **oxycodone** | PM ? UM? |
| | **paroxetine** | UM |
| | **pimozide** | PM, IM |
| | **propafenone** | PM, IM, UM |
| | tamoxifen | PM, IM |
| | **tramadol** | PM, IM, UM |
| | **venlafaxine** | PM, IM, UM |
| | **zuclopenthixol** | PM, IM, UM |
| *CYP3A5* | tacrolimus | Homozygote expressor, heterozygote expressor |
| *DPYD* | capecitabine/**fluorouracil** | Systemic: GAS 0, 0.5, 1, 1.5; Topical: GAS 0 |
| | tegafur | Systemic: GAS 0, 0.5, 1, 1.5 |
| *SLCO1B1* | **atorvastatin** | TC, TT |
| | **simvastatin** | TC, TT |
| *TPMT* | azathioprine/mercaptopurine | PM, IM |
| | thioguanine | PM, IM |
| *VKORC1* | **acenocoumarol** | AA |
| | **fenprocoumon** | AA |

Drugs primarily prescribed in primary care are bolded

Department Clinical Pharmacology and Toxicology
postzone L-00-P

sender
visiting address
Phone fax
e-mail

to [PHARMACIST NAME]

cc [GENERAL PRACTITIONER NAME]

Date 26 november 2014
subject Genotype results for patiënt XXXX

Dear Colleague,

Through this letter I would like to inform you that patient [PATIENT NAME], born [DATE OF BIRTH], participates in the IP3 study and has been genotyped for 8 genes that are related to the effectiveness and toxicity of drugs. This letter contains the results of the genotyping and the interpretation of the genotypes.

.

**Patient:** [PATIENT NAME]
**Date of Birth:** XX-XX-XXXX
**General Practitioner:** [GENERAL PRACTITIONER NAME]
**Pharmacy:** [PHARMACY NAME]
**Studynumber:** IP3-XXX

Method
The DNA was isolated from saliva using the Oragene kit and then analyzed with the Affymetrix DMET array according to the manufacturer's protocol. In addition, the number of CYP2D6 copies has been determined with a Taqman genotyping test. The translation from genotype to phenotype was carried out in accordance with the guidelines drawn up by the pharmacogenetics working group of the KNMP.

Results

| Gene | Tested variant alleles | Patient genotype | Predicted Phenotype |
|---|---|---|---|
| *CYP2C9* | *2, *3 | *1/*2 | **Intermediate metabolizer** |
| *CYP2C19* | *2, *17 | *1/*1 | Extensive metabolizer |
| *CYP2D6* | 22 allelen* | *4/*5 | **Poor metabolizer** |
| *CYP3A5* | *3, *6 | *3/*3 | Non-expressor |
| *DYPD* | *2A, *13 | *1/*1 | Extensive metabolizer |
| *SLCO1B1* | 521T>C | 521 TC | **Decreased function** |
| *TPMT* | *2, *3C, *3B | *1/*1 | Extensive metabolizer |
| *VKORC1* | 1173C>T | 1173 CC | Normal sensitivity |

Interpretation of abberant genotypes

The CYP2C9 * 1 / * 2 genotype leads to the intermediate metabolizer phenotype. Persons with this phenotype have a reduced metabolic capacity of the enzyme CYP2C9 and an increased risk of side effects and efficacy in drugs metabolised by CYP2C9

The CYP2D6 * 4 / * 5 genotype leads to the poor metaboliser phenotype. Individuals with this phenotype have a greatly reduced or absent metabolic capacity of the enzyme CYP2D6 and a greatly increased risk of side effects and efficacy in drugs metabolised by CYP2D6.

The SLCO1B1 521 TC genotype leads to a reduced transport activity of statins from the portal vein to the liver cells. As a result, the plasma concentration of statins and thereby the risk of myopathy can increase.

Recommendation for drug of enrolment

The recommendation the KNMP pharmacogenetics working group for the use of atorvastatin in patients with the SLCO1B1 521 TC genotype is:

1. If this patient has additional risk factors for statin-induced myopathy *:
    1.1. to choose an alternative to atorvastatin.
    Rosuvastatin and pravastatin are similarly affected by SLCO1B1 polymorphisms, but are not affected by CYP3A4 inhibitors such as amiodarone, verapamil and diltiazem.
    Fluvastatin is not affected by SLCO1B1 polymorphisms and CYP3A4 inhibitors.
    1.2. or if an alternative is not possible: advise the patient to contact muscle complaints.
    * Use of CYP3A4 inhibitors, colchicine, fusidic acid and gemfibrozil as co-medication.
2. If this patient has no significant additional risk factors for statin-induced myopathy:
Advise the patient to contact him if you have a muscle complaint.

I request you to record the patient's genotypes found as a contraindication in your electronic prescribing system. A notification will automatically follow if there is a relevant gene-drug interaction.

I hope to have informed you sufficiently. If you have any questions, you can always contact us by email or telephone.

Kind regards,

[NAME CLINICAL PHARMACIST]

**Supplementary Figure S1.** Example report sent to physicians and pharmacists.

**Supplementary Table 3.** Pre-defined drug-gene associated adverse drug reactions based on literature underlying the DPWG.

| Drug | Gene | Phenotype | Increased risk of adverse event | Effect measure* |
|---|---|---|---|---|
| Amitriptyline | CYP2D6 | PM | The genetic polymorphism leads to a reduced metabolic capacity of CYP2D6, causing an increase in the plasma concentrations of amitriptyline and its active metabolite nortriptyline and a decrease in the plasma concentrations of the active metabolites E-10-OH-amitriptyline and E-10-OH-nortriptyline. [1]<br>The side effects are correlated to the plasma concentration of nortriptyline.<br>The hydroxy-metabolites may be cardiotoxic. [1]<br>Theoretically, the risk of side effects such as dry mouth, constipation, dizziness, sedation, reduction of sexual functions and perspiration is increased with high plasma concentration of nortriptyline. [1] | Studies found an increase of 30-69% of the plasma concentration amitriptyline plus nortriptyline. [1]<br>PMs did not have excessive side effects. [2] |
| Amitriptyline | CYP2D6 | IM | The genetic polymorphism leads to a reduced metabolic capacity of CYP2D6, causing an increase in the plasma concentrations of amitriptyline and its active metabolite nortriptyline and a decrease in the plasma concentrations of the active metabolites E-10-OH-amitriptyline and E-10-OH-nortriptyline. [1]<br>The side effects are correlated to the plasma concentration of nortriptyline. | In a study an increase of the percentage of patients with substantial side effects was found by a factor of 6. For the subgroup of patients without co-medication affecting CYP2D6, the percentage increased by a factor of 16. This study found for patients with phenotypes IM versus EM + UM an increase in the percentage of patients with substantial side effects from 12.1% to 76.5% (S by 523%) The same for patients without CYP2D6-relevant comedication: from 4.2% to 69.2% (S by 1548%). [3] |

| | | | | |
|---|---|---|---|---|
| | | | The hydroxy-metabolites may be cardiotoxic. [1] Theoretically, the risk of side effects such as dry mouth, constipation, dizziness, sedation, reduction of sexual functions and perspiration is increased with high plasma concentration of nortriptyline. [1] | |
| Amitriptyline | CYP2D6 | UM | The genetic polymorphism leads to an increased metabolic capacity of CYP2D6, which may decrease the plasma concentrations of amitriptyline and its active metabolite nortriptyline and increase the plasma concentrations of the active metabolites E-10-OH-amitriptyline and E-10-OH-nortriptyline. [1] Result: Possible failure of therapy due to decreased plasma concentrations of amitriptyline and nortriptyline and an increase in the plasma concentration of the potentially cardiotoxic, active hydroxy-metabolites. [1] | A study found a decrease of the plasma concentration of amitriptyline plus nortriptyline with 20% (non-significant). [4] |
| Atomoxetine | CYP2D6 | PM | The genetic variation increases the plasma concentration of atomoxetine and thus the risk of side effects (such as loss of appetite, vomiting, abdominal pain, constipation, insomnia, early awakening, drowsiness, irritability, pupil dilation, itching, dry mouth, urinary retention, erectile dysfunction, excessive sweating, increase of heart rate, increase in diastolic blood pressure palpitations, | Results vary from no reduced appetite to an increase in the incidence of reduced appetite with 42% or OR = 2.0. The incidence of tremor increased by 364% and the incidence of insomnia by 54% or OR = 2.1.[4,9] In a study with 117 adult PM, PMs had a higher risk of urinary retention (OR = 9.1), an erectile dysfunction (OR = 3.1), a dry mouth (OR = 2.2), an increase in diastolic blood pressure ( OR = 2.2), excessive sweating (OR = 2.0) and an increase in heart rate (OR = 1.7). Sedation, depression, early awakening, pruritus and |

| | | | | |
|---|---|---|---|---|
| | | | dizziness, increased systolic blood pressure, tremor and sedation).[5–8] | mydriasis are also more common in PMs. [5]<br>In 131 healthy male PMs, a dose of 60 mg 2x daily resulted in a statistically significant but not clinically significant increase in the QT interval. For the increase relative to placebo, the upper limit of the confidence interval was less than 10 msec. There were no subjects at any time with a corrected QT interval greater than 500 msec or an increase in the corrected QT interval by more than 60 msec with respect to pre-treatment. [7]<br>The results for the incidence of discontinuation of therapy due to side effects vary from no difference, an increase by 3-50% or a decrease after 6 months by 100%. [5] |
| Atomoxetine | CYP2D6 | IM | The genetic variation increases the plasma concentration of atomoxetine and can therefore reduce the dose requirement. [5]<br>Side effects related to high atomoxetine levels: dry mouth, sleep disturbances, dizziness, nausea and abdominal pain. [6,10] | The plasma concentration of atomoxetine is a factor 2-3 times higher for IM than for EM at the same dose.<br>Results range from no difference in frequency, severity and nature of the side effects to an increase in the risk of a sleep disorder (OR = 1.7) or dry mouth (OR = 1.6). IM were not overrepresented in patients who did not finish treatment and the mean dose was similar for IM and EM/UM. [6]<br>One study found that of 10 patients who had side effects and/or a late response at normal dosing, 6 were IM. In the two IMs where the dose was reduced (up to 1.14 mg/kg per day and 0.42 mg/kg per day), this led to maintenance of efficacy and decrease in side effects. [8] |
| Atomoxetine | CYP2D6 | UM | The genetic variation leads to an increased conversion of atomoxetine into the active metabolite 4-hydroxyatomoxetine, which has a much lower plasma concentration. Because the | - |

| | | | | |
|---|---|---|---|---|
| | | | plasma concentration of the active substances decreases as a result, the gene variation can lead to a reduced effectiveness. | |
| Atorvastatin | SLCO1B1 | 521TC | The genetic polymorphism can lead to a reduced transport of atorvastatin to the liver. This may increase the plasma concentration of atorvastatin and thus the risk of myopathy. [11] | Myopathy<br>Results range from no significant effect of the genetic polymorphism on the risk of myopathy or muscle complaints (two studies with 143-146 atorvastatin users and two case-control study with 10-13 cases) to an association of the 521C allele with intolerance or muscle complaints with OR = 2.7 (case-control study with 46 case). [12]<br>In one case involving two related patients with atorvastatin-induced muscle pain, one patient had genotype 521CC and the other genotype 521TC. [11]<br><br>Cholesterol reduction<br>In two studies, there was no difference in the decrease of LDL cholesterol. [11] |
| Atorvastatin | SLCO1B1 | 521CC | The genetic polymorphism can lead to a reduced transport of atorvastatin to the liver. This may increase the plasma concentration of atorvastatin and thus the risk of myopathy. [11] | Myopathy<br>Results range from no significant effect of the genetic polymorphism on the risk of myopathy or muscle complaints (two studies with 143-146 atorvastatin users and two case-control study with 10-13 cases) to an association of the 521C allele with intolerance or muscle complaints with OR = 2.7 (case-control study with 46 case). [12–16]<br>In one case involving two related patients with atorvastatin-induced muscle pain, one patient had genotype 521CC and the other genotype 521TC. [11]<br><br>Cholesterol reduction<br>In two studies, there was no difference in the decrease of LDL cholesterol. [17,18] |
| Citalopram | CYP2C19 | PM | This gene variation leads to an increase in plasma concentrations of citalopram. This causes a hogher risk of | A study found a 3.0% greater QTc interval for a group of 16 IM and 1 PM. The study found no difference for this group in the median dose and the percentage of |

| | | | | |
|---|---|---|---|---|
| | | | QT-prolongation and torsade de pointes. [19] | patients with a dose higher than 40 mg/day. [20]<br>Two studies found no difference in the occurrence of side effects. A study with new-borns found no difference in severity of serotonergic symptoms after mother's citalopram use for a group of 4 IM and 1 PM. [21]<br>For the probability of remission, the effect varies from no difference to an increase of 48%. [22] |
| Citalopram | CYP2C19 | IM | This gene variation leads to an increase in plasma concentrations of citalopram. This causes a higher risk of QT prolongation and torsade de pointes. [19]<br>The relationship between plasma concentration and efficacy and side effects has not been established. The risk of induction of QT prolongation and torsade de pointes by citalopram is dose-dependent and therefore plasma concentration-dependent. [19] | A study with 16 IM found a trend for a 2.4% larger QTc interval. The study found a significant increase in the QTc interval for a group of 16 IM and 1 PM and no difference in the median dose and the percentage of patients with a dosage higher than 40 mg/day. [20]<br>A study with 25 IM found no difference in the occurrence of side effects. [23] A study with new-borns found no difference in severity of serotonergic symptoms after mother's citalopram use for a group of 4 IM and 1 PM. For IM + PM, the results for the probability of tolerance vary from no difference in the validation study to a decrease. [21]<br>A study with 298 IM found no difference in the chance of remission. The same study found no association between set dose and genotype. [22] |
| Citalopram | CYP2C19 | UM | The gene variation increases the conversion of escitalopram to a low active substance. However, no significant effect on plasma concentration of citalopram, tolerance and response has been demonstrated. [19] | A study with 60 UM found no difference in the likelihood of tolerance and remission. [22]<br>Two studies found no difference in set dosage.<br>A study with 18 UM found no significant increase in the percentage of patients with plasma concentrations below the therapeutic range. [4] |
| Escitalopram | CYP2C19 | PM | The gene variation leads to an increase in the plasma concentration of escitalopram. | A study found no increase in the QTc interval for a group of 1 PM and 21 IMs. However, the IM + PM group and the EM |

| | | |
|---|---|---|
| | This increases the risk of QT prolongation and torsade de pointes. [24]<br>Side effects related to higher escitalopram levels are dry mouth, dizziness and diarrhoea. [25] | group were not comparable. The percentage of women was significantly lower for IM + PM. Women had a 3.7% higher QTc interval than men. In addition, the percentage of patients with a CYP2C19 substrate, inhibitor or inducer was significantly higher for IM + PM. There was a trend for a 2.8% higher QTc interval when using this co-medication. [20] A study with 6 PM found no difference between the genotypes in adverse events and in the percentage of patients who discontinued in the study. [25] Another study found no difference in neurological, psychological and 'other' side effects for a group of 23 IM + PM after 1 week. The score for autonomic side effects, such as sweating and gastrointestinal complaints, was reduced after 1 week, but this is probably not clinically relevant. [26]<br>There was no difference in the dose adjusted according to side effects and effect.<br>Three studies found no difference in response to depression (one with 16 PM, one with 9 PM and one with 23 IM + PM). [25–27] A study with 1 PM found no difference in response to peripheral neuropathy. [28] For a group with 22 IMs and 1 PM, a study found no difference in response to autism spectrum disorder. [29]<br><br>There was no association of escitalopram plasma concentration found with the number of side effects or the occurrence of side effects. The adverse events dry mouth was increased with high escitalopram plasma concentration (OR = 1.48). The side effect diarrhoea occurred less frequently with higher ratios of desmethylescitalopram/escitalopram (OR = 0.60; S). [25] |

| | | | | |
|---|---|---|---|---|
| Escitalopram | CYP2C19 | IM | The gene variation leads to an increase in the plasma concentration of escitalopram. This increases the risk of QT prolongation and torsade de pointes. [24]<br>Side effects related to higher escitalopram levels: dry mouth, dizziness and diarrhoea. [25] | A study found no increase in the QTc interval for a group of 1 PM and 21 IMs. However, the IM + PM group and the EM group were not comparable. The percentage of women was significantly lower for IM + PM. Women had a 3.7% higher QTc interval than men. In addition, the percentage of patients with a CYP2C19 substrate, inhibitor or inducer was significantly higher for IM + PM. There was a trend for a 2.8% higher QTc interval when using this co-medication. [20]<br>A 94 IM study found no difference between the genotypes in adverse reactions and in the percentage of patients who discontinued the study. Another study found no difference in neurological, psychological and 'other' side effects for a group of 23 IM + PM after 1 week. The score for autonomic side effects, such as sweating and gastrointestinal complaints, was reduced after 1 week, but this is probably not clinically relevant.<br>There was no difference in the dose adjusted according to side effects and effect. A study with 116 IM found no difference in response to depression. Another study found no difference for a group of 23 IM + PM. [26] A study with 7 IM found no difference in response to peripheral neuropathy. [28] For a group with 22 IMs and 1 PM, a study found no difference in response to autism spectrum disorder. [29]<br>There was no association of escitalopram plasma concentration found with the number of side effects or the occurrence of side effects. The adverse events dry mouth was increased with high escitalopram plasma concentration (OR = 1.48). The side effect diarrhoea occurred less frequently with higher ratios of |

| | | | | |
|---|---|---|---|---|
| | | | | desmethylescitalopram/escitalopram (OR = 0.60; S). [25] |
| Escitalopram | CYP2C19 | UM | NO action is required with this gene-drug interaction.<br>The gene variation increases the conversion of escitalopram to a low active substance. However, this does not lead to a reduced effect, a need for a higher dose or an increase in side effects. [24]<br><br>High desmethylescitalopram plasma concentration increased the occurrence of vertigo (OR = 1.56; S). [25] | A study with 28 UM found no difference in response to depression. [25] A study with 2 UM found no difference in response to peripheral neuropathy. [28] For a group with 9 UMs and 17 times *1/*17, a study found no difference in response to autism spectrum disorder. [29]<br>The first and last study also found no effect of the genotype on the final dose. The latter study found no difference in the rate of dose increase during the whole 6 week treatment period, but found a lower rate of dose increase in the fourth, fifth and sixth week after the start of treatment. [29]<br>Two studies with a total of 27 UM found no difference in side effects. [24,25] |
| Clomipramine | CYP2D6 | IM | The genetic polymorphism leads to a reduced metabolic capacity of CYP2D6. As a result, the plasma concentrations of clomipramine and the active metabolite may increase and those of the potentially cardiotoxic hydroxy-metabolites may decrease. Side effects include dry mouth, constipation, dizziness, sedation, reduction of sexual functions and transpiration. [30] | In a study, an increase in the percentage of patients with adverse events was found with a factor of 1.9. [31] |
| Clomipramine | CYP2D6 | PM | The genetic polymorphism leads to a reduced metabolic capacity of CYP2D6. As a result, the plasma concentrations of clomipramine and the active metabolite may increase and those of the potentially cardiotoxic hydroxy-metabolites may decrease. | In two cases, side effects were observed. [32] The side effects disappeared in a case after lowering the clomipramine dose. [33] As a result, plasma concentrations of clomipramine and N-desmethylclomipramine reached the therapeutic range.<br>There was an increase in plasma concentration of clomipramine + desmethylclomipramine by 88-199%. [4,34] |

| | | | | |
|---|---|---|---|---|
| | | | Side effects include dry mouth, constipation, dizziness, sedation, reduction of sexual functions and perspiration. [30] | For the plasma concentration of clomipramine, the results vary of a decrease by 34% to an increase of 185%. [34,35]<br>After single administration, clomipramine clearance decreased by 43% and half-life increased by 21%. [34] |
| Clomipramine | CYP2D6 | UM | The genetic polymorphism leads to an increased metabolic capacity of CYP2D6. As a result, the plasma concentrations of clomipramine and the active metabolite may decrease and those of the potentially cardiotoxic hydroxyl-metabolites may increase.<br>The inactive hydroxy-metabolites may be cardiotoxic. These are formed to an increased extent at UM and at dose increases. The hydroxy-metabolites accumulate in severe renal dysfunction.<br>The active metabolite desmethylclomipramine does not have serotonin reuptake activity. The metabolite therefore does not appear to contribute to the treatment of obsessive-compulsive disorder and other anxiety disorders. The metabolite does contribute to toxicity and treatment of depression. [30] | In two cases with non-response, increased plasma concentrations due to dose escalation or CYP2D6 inhibition led to recovery of the problem. [36,37] The dose increase involved an increase of 150–300 mg/day. Other reports of dose increase at UM are not known.<br>On theoretical grounds, the risk of adverse reactions due to the possible cardiotoxic hydroxy-metabolites increases with higher plasma concentrations. [30] |
| Nortriptyline | CYP2D6 | PM | The genetic polymorphism leads to a reduced metabolic capacity of CYP2D6 which may increase the plasma concentration of nortriptyline. [38] | In a study no significant change in the percentage of patients with side effects was found after 6 weeks use of nortriptyline. [40]<br>In a case, side effects were observed, which disappeared after normalization of the plasma concentration of nortriptyline and |

| | | | | |
|---|---|---|---|---|
| | | | Side effects include dry mouth, constipation, dizziness, nervousness and tinnitus (tinnitus), instability of the knees, drowsiness, inertia, anxiety, agitation, hypotension and fatigue. [39] | E-10-hydroxynortriptyline by dose reduction. [41] The plasma concentration and AUC of nortriptyline increase by 146% and 232%, respectively. Oral clearance decreases with 62%. [42–44] |
| Nortriptyline | CYP2D6 | IM | The genetic polymorphism leads to a reduced metabolic capacity of CYP2D6, which may increase the plasma concentration of nortriptyline. [38] Anticholinergic adverse reactions (dry mouth, constipation, dizziness) reported in 1 case, disappeared with dose reduction. In another case, nervousness and tinnitus (ringing in the ears), instability of the knees, drowsiness, slowness, anxiety, agitation and side effects have been reported. [39] | The plasma concentration and AUC of nortriptyline increase by 35–123% and 86-179%, respectively. [42,43,45,46] Clearance decreases by 31% -57%. [44,46] The dose decreases to 70% of the dose at EM. [38] |
| Nortriptyline | CYP2D6 | UM | The genetic polymorphism leads to an increased metabolic capacity of CYP2D6, which may decrease the plasma concentration of nortriptyline and increase the plasma concentration of the active metabolite E-10-OH-nortriptyline. E-10-hydroxynortriptyline is about half as potent as the parent compound in inhibiting norepinephrine uptake. It has a much lower anticholinergic activity than nortriptyline and is associated with cardiotoxicity. [38] | On theoretical grounds, the risk of cardiotoxic adverse reactions is increased with an increased plasma concentration of E-10-hydroxynortriptyline and the risk of reduced effectiveness of therapy is increased with a reduced plasma concentration of nortriptyline.[15] In studies, the AUC of nortriptyline was reduced by 23-41% and the oral clearance increased by 85%. [44,47] At 13 functional alleles: for nortriptyline increase clearance by 62% -315% and decrease half-life by 12%. [43,44] |
| Simvastatin | SLCO1B1 | 521TC | The genetic polymorphism can lead to a reduced transport of | Myopathy |

| | | | | |
|---|---|---|---|---|
| | | | simvastatin to the liver. This may increase the plasma concentration of simvastatin and thus the risk of myopathy. [48] | The risk of myopathy was increased. The increase of myopathy seems to increase with the simvastatin dose.<br>In a study with simvastatin 80 mg/day, the OR for myopathy with creatine kinase was higher than 3 or 10 times the upper limit of normal 4.5 (95% CI [2.6-7.7]) per 521C allele. The calculated cumulative myopathy risk was 3% for 521CT versus 0.6% for 521TT. The OR for myopathy per 521C allele was 2.6 (95% CI [1.3-5.0]) for simvastatin 40 mg/day. [49]<br>In a study with simvastatin 30 mg/day on average there was no significant increase in the risk of myopathy with creatine kinase higher than 10 times the upper limit of normal for (521TC + 521CC). [15] In a study with simvastatin 20 mg/day followed by 80 mg/day, the percentage of patients who either discontinued the study prematurely due to an adverse reaction or developed myalgia or muscle cramps or increased creatine kinase to more than 3 times the upper limit of normal had increased by a factor of 2.2 for (521TC + 521CC). [14]<br><br>Cholesterol reduction<br>In three studies there was no difference in the decrease of LDL cholesterol. [50–52] In one study, the decrease in LDL-cholesterol decreased by 3.2% per 521C allele. [48] |
| Simvastatin | SLCO1B1 | 521CC | The genetic polymorphism leads to a reduced transport of simvastatin to the liver. This increases the plasma concentration of simvastatin and thus the risk of myopathy.[48] | The risk of myopathy was increased. The increase of myopathy seems to increase with the simvastatin dose.<br>In a study with simvastatin 80 mg/day, the OR for myopathy with creatine kinase was higher than 3 or 10 times the upper limit of normal 4.5 (95% CI [2.6-7.7]) per 521C allele. The calculated cumulative myopathy risk was 3% for 521CT versus 0.6% for 521TT. The OR for myopathy per |

| | | | | |
|---|---|---|---|---|
| | | | | 521C allele was 2.6 (95% CI [1.3-5.0]) for simvastatin 40 mg/day. [49]<br>In a study with simvastatin 30 mg/day on average there was no significant increase in the risk of myopathy with creatine kinase higher than 10 times the upper limit of normal for (521TC + 521CC). [15] In a study with simvastatin 20 mg/day followed by 80 mg/day, the percentage of patients who either discontinued the study prematurely due to an adverse reaction or developed myalgia or muscle cramps or increased creatine kinase to more than 3 times the upper limit of normal had increased by a factor of 2.2 for (521TC + 521CC). [14]<br><br>Cholesterol reduction<br>In three studies there was no difference in the decrease of LDL cholesterol. [50–52] In one study, the decrease in LDL-cholesterol decreased by 3.2% per 521C allele. [48] |
| Venlafaxine | CYP2D6 | PM | The genetic polymorphism leads to a reduced metabolic capacity of CYP2D6. As a result, the plasma concentration of venlafaxine may increase and that of the active metabolite O-desmethylvenlafaxine may decrease. There are indications that the effectiveness of venlafaxine is reduced in patients with this genetic polymorphism. [53]<br>Side effects related to elevated venlafaxine levels: elevation of alkaline phosphatase levels, sweating, insomnia, dry mouth, increased appetite, drowsiness, diminished effect, nausea, anxiety, palpitations, | The results of a decrease in effectiveness vary to no difference in efficacy with respect to EM + IM in patients with depression. In a study with 3 PM there was 100% non-response. [53]<br>In obsessive compulsive disorder, there was no difference in effectiveness. [56]<br>For side effects, the results vary from no difference to an increase in the number of side effects by 369% (increase in the number of side effects per patient from 0.49 to 2.3 (S by 369%). [54,57] There is virtually no effect on the sodium concentration (decrease by 3%). [54]<br>Cardiac adverse reactions (syncope, palpitations, dizziness) have been reported. [58]<br><br>A study found an statistically significant increase in the number of patients with high alkaline phosphatase levels by a |

| | | | | |
|---|---|---|---|---|
| | | | vomiting and diarrhoea. [53–55]<br>Cardiac events (syncope, palpitations, dizziness) have been reported.<br>Venlafaxine is possibly cardiotoxic.<br>In one study, reduced efficacy in depression was found in patients with an elevated ratio of venlafaxine/active metabolite (PM). [53] | factor of 20.5 (from 0.2% to 4.1%) when comparing PM versus EM+IM+UM. The number of patients which has sweating as side-effect was statistically significant increased by a factor of 1.9 (from 13.3% to 24.5%) and the number of patients with insomnia increased statistically significant by a factor of 1.7 (from 22.4% to 38.8%). [57] |
| Venlafaxine | CYP2D6 | IM | The genetic polymorphism leads to a reduced metabolic capacity of CYP2D6. As a result, the plasma concentration of venlafaxine may increase and that of the active metabolite O-desmethylvenlafaxine may decrease. [53]<br>Venlafaxine is possibly cardiotoxic.<br>In one study, reduced efficacy in depression was found in patients with an elevated ratio of venlafaxine/active metabolite (PM).<br>Cardiac events (syncope, palpitations, dizziness) have been reported.<br>Side effects related to elevated venlafaxine levels: elevation of alkaline phosphatase levels, sweating, insomnia, dry mouth, increased appetite, drowsiness, diminished effect, nausea, anxiety, palpitations, vomiting and diarrhoea. [53-55] | For venlafaxine + O-desmethyl venlafaxine, AUC increases by 14-17% and plasma concentration by 1-22%. [54,59,60] The ratio of plasma concentrations of O-desmethylvenlafaxine/venlafaxine decreases by 52-66%. [54,60] The decrease in the ratio is mainly caused by an increase in the plasma concentration of venlafaxine. [53] |

| | | | | |
|---|---|---|---|---|
| Venlafaxine | CYP2D6 | UM | The genetic polymorphism leads to an increased metabolic capacity of CYP2D6. As a result, the plasma concentration of venlafaxine may decrease and that of the active metabolite O-desmethylvenlafaxine may increase. [53] | In one study, the number of adverse events did not significantly decrease by 39% (0.49 to 0.3) and there was no difference in therapeutic efficacy (both 1.7 points). [54]<br>In another study there was no effect on the sodium concentration. [53] |
| Doxepin | CYP2D6 | IM | The genetic polymorphism leads to a reduced metabolic capacity of CYP2D6, which may increase plasma concentrations of doxepin and nordoxepin. [61]<br>On theoretical grounds, the risk of side effects increases when plasma concentrations of doxepin and nordoxepin increase. [61] | In single-dose administration of 75 mg doxepin, the AUC of doxepin + nordoxepin increased by 19% and the oral clearance of doxepin decreased by 42%. [62] |
| Doxepin | CYP2D6 | UM | Genetic polymorphism leads to increased metabolic capacity of CYP2D6, which may decrease plasma concentrations of doxepin and nordoxepin and increase plasma concentrations of the hydroxy-metabolites. [61]<br>On theoretical grounds, the risk of reduced effectiveness of therapy increases when plasma concentrations of doxepin and nordoxepin decrease. [61] | The AUC of doxepin + nordoxepin was reduced by 55% (from 1061 to 479 nmol.h/L). [62] |
| Doxepin | CYP2D6 | PM | The genetic polymorphism leads to a reduced metabolic capacity of CYP2D6, which may increase plasma concentrations of doxepin and nordoxepin. [61] | The AUC of doxepin + nordoxepin increases by 116-190% with a single administration. [62,63]<br>For multiple-dose administration, results vary from a decrease of 12% in plasma concentration of doxepin (a case) to an increase in the frequency of PMs from 0-50% in patients with high plasma |

| | |
|---|---|
| | concentrations versus patients with low to normal plasma concentrations. |

*The risk on a side effect when compared with EM; PM, poor metabolizer; IM, intermediate metabolizer; UM, ultrarapid metabolizer; EM, extensive metabolizer.

**References Appendix 1**


1. Kennisbank, Koninklijke Nederlandse Maatschappij ter bevordering der Pharmacie (KNMP), Version 3.0.5. Farmacogenetica. AMITRIPTYLINE CYP2D6 IM-PM-UM. Available online: https://kennisbank.knmp.nl/article/farmacogenetica/intro.html (accessed on 29 March 2019).
2. Baumann, P.; Jonzier-Perey, M.; Koeb, L.; Küpfer, A.; Tinguely, D.; Schöpf, J. Amitriptyline pharmacokinetics and clinical response: II. Metabolic polymorphism assessed by hydroxylation of debrisoquine and mephenytoin. *Int. Clin. Psychopharmacol.* **1986**, *2*, 102–112.
3. Steimer, W.; Zöpf, K.; von Aemlunxen, S.; Preiffer, H.; Bachofer, J.; Popp, J.; et al. Amitriptyline or not, that is the question: Pharmacogenetic testing of CYP2D6 and CYP2C19 identifies patients with low or high risk for side effects in amitriptyline therapy. *Clin. Chem.* **2005,** *2*, 376–385.
4. de Vos, A.; van der Weide, J.; Loovers, H.M. Association between CYP2C19*17 and metabolism of amitriptyline, citalopram and clomipramine in Dutch hospitalized patients. *Pharmacogenomics J.* **2011**, *5*, 359–367. doi: 10.1038/tpj.2010.39.
5. Kennisbank, Koninklijke Nederlandse Maatschappij ter bevordering der Pharmacie (KNMP), Version 3.0.5. Farmacogenetica. ATOMOXETINE CYP2D6 PM-IM-UM. Available online: https://kennisbank.knmp.nl/article/farmacogenetica/intro.html (accessed on 29 March 2019).
6. Fijal, B.A.; Guo, Y.; Li, S.G.; Ahl, J.; Goto, T.; Tanaka, Y.; Nisenbaum, L.K.; Upadhyaya, H.P. CYP2D6 predicted metabolizer status and safety in adult patients with attention-deficit hyperactivity disorder participating in a large placebo-controlled atomoxetine maintenance of response clinical trial. *J. Clin. Pharmacol.* **2015**, *55*, 1167–1174.
7. Loghin, C.; Haber, H.; Beasley, C.M.Jr.; Kothare, P.A.; Kauffman, L.; April, J.; et al. Effects of atomoxetine on the QT interval in healthy CYP2D6 poor metabolizers. *Br. J. Clin. Pharmacol.* **2013**, *2*, 538–49. doi: 10.1111/j.1365-2125.2012.04382.x.
8. ter Laak, M.A.; Temmink, A.H.; Koeken, A.; van 't Veer, N.E.; van Hattum, P.R.; Cobbaert, C.M. Recognition of impaired atomoxetine metabolism because of low CYP2D6 activity. *Pediatr Neurol.* **2010**, *3*, 159–62. doi: 10.1016/j.pediatrneurol.2010.04.004.
9. Michelson, D.; Read, H.A.; Ruff, D.D.; Witcher, J.; Zhang, S.; McCracken, J. CYP2D6 and clinical response to atomoxetine in children and adolescents with ADHD. *J. Am. Acad. Child Adolesc. N.a.* **2007**, *46*, 242–251.
10. Cui, Y.M.; Teng, C.H.; Pan, A.X.; Yuen, E.; Yeo, K.P.; Zhou, Y.; Zhao, X.; Long, A.J.; Bangs, M.E.; Wise, S.D. Atomoxetine pharmacokinetics in healthy Chinese subjects and effect of the CYP2D6*10 allele. *Br. J. Clin. Pharmacol.* **2007**, *64*, 445–449.
11. Kennisbank, Koninklijke Nederlandse Maatschappij ter bevordering der Pharmacie (KNMP), Version 3.0.5. Farmacogenetica. ATORVASTATINE SLCO1B1 521TC-521CC. Available online: https://kennisbank.knmp.nl/article/farmacogenetica/intro.html (accessed on 29 March 2019).
12. Puccetti, L.; Ciani, F.; Auteri, A. Genetic involvement in statins induced myopathy. Preliminary data from an observational case-control study. *Atherosclerosis* **2010**, *1*, 28–29. doi: 10.1016/j.atherosclerosis.2010.02.026.
13. Santos, P.C.; Gagliardi, A.C.; Miname, M.H.; Chacra, A.P.; Santos, R.D.; Krieger, J.E.; Pereira, A.C. SLCO1B1 haplotypes are not associated with atorvastatin-induced myalgia in Brazilian patients with familial hypercholesterolemia. *Eur. J. Clin. Pharmacol.* **2012**, *3*, 273–9. doi: 10.1007/s00228-011-1125-1.
14. Voora, D.; Shah, S.H.; Spasojevic, I.; Ali, S.; Reed, C.R.; Salisbury, B.A.; Ginsburg, G.S. The SLCO1B1*5 genetic variant is associated with statin- induced side effects. *J. Am. Coll. Cardiol.* **2009**, *17*, 1609–1616. doi: 10.1016/j.jacc.2009.04.053.
15. Brunham, L.R.; Lansberg, P.J.; Zhang, L.; Miao, F.; Carter, C.; Hoving, G.K.; Visscher, H.; Jukema, J.W.; Stalenhoef, A.F.; Ross, C.J.; et al. Differential effect of the rs4149056 variant in SLCO1B1 on myopathy associated with simvastatin and atorvastatin. *Pharmacogenomics J.* **2012**, *3*, 233–7. doi: 10.1038/tpj.2010.92.
16. Hermann, M.; Bogsrud, M.P.; Molden, E.; Asberg, A.; Mohebi, B.U.; Ose, L.; Retterstøl, K. Exposure of atorvastatin is unchanged but lactone and acid metabolites are increased several- fold in patients with atorvastatin-induced myopathy. *Clin. Pharmacol. Ther.* **2006**, *6*, 532–9.
17. Rodrigues, A.C.; Perin, P.M.S.; Purim, S.G.; Silbiger, V.N.; Genvigir, F.D.V.; Willrich, M.A.V.; Arazi, S.S.; Luchessi, A.D.; Hirata, M.H.; Bernik, M.M.S.; et al. Pharmacogenetics of OATP Transporters Reveals That SLCO1B1 c.388A>G Variant Is Determinant of Increased Atorvastatin Response. *Int. J. Mol. Sci.* **2011**, *12*, 5815–5827.

18. Mega, J.L.; Morrow, D.A.; Brown, A.; Cannon, C.P.; Sabatine, M.S. Identification of genetic variants associated with response to statin therapy. *Arter. Thromb. Vasc. Boil.* **2009**, *29*, 1310–1315.
19. Kennisbank, Koninklijke Nederlandse Maatschappij ter bevordering der Pharmacie (KNMP), Version 3.0.5. Farmacogenetica. CITALOPRAM CYP2C19 IM-PM-UM. Available online: https://kennisbank.knmp.nl/article/farmacogenetica/intro.html (accessed on 29 March 2019).
20. Kumar, Y.; Kung, S.; Shinozaki, G. CYP2C19 variation, not citalopram dose nor serum level, is associated with QTc prolongation. J. Psychopharmacol. **2014**, *12*, 1143–1148. doi: 10.1177/0269881114543720
21. Hilli, J.; Heikkinen, T.; Rontu, R.; Lehtimäki, T.; Kishida, I.; Aklillu, E.; Bertilsson, L.; Vahlberg, T.; Laine, K. MAO-A and COMT genotypes as possible regulators of perinatal serotonergic symptoms after in utero exposure to SSRIs. *Eur. Neuropsychopharmacol.* **2009**, *5*, 363–370. doi: 10.1016/j.euroneuro.2009.01.006.
22. Mrazek, D.A.; Biernacka, J.M.; O'Kane, D.J.; Black, J.L.; Cunningham, J.M.; Drews, M.S.; Snyder, K.A.; Stevens, S.R.; Rush, A.J.; Weinshilboum, R.M. CYP2C19 variation and citalopram response. *Pharmacogenet. Genomics.* **2011**, *1*, 1–9.
23. Yin, O.Q.; Wing, Y.-K.; Cheung, Y.; Wang, Z.-J.; Lam, S.-L.; Chiu, H.F.; Chow, M.S. Phenotype-genotype Relationship and Clinical Effects of Citalopram in Chinese Patients. *J. Clin. Psychopharmacol.* **2006**, *26*, 367–372.
24. Kennisbank, Koninklijke Nederlandse Maatschappij ter bevordering der Pharmacie (KNMP), Version 3.0.5. Farmacogenetica. ESCITALOPRAM CYP2C19 UM-IM-PM. Available online: https://kennisbank.knmp.nl/article/farmacogenetica/intro.html (accessed on 29 March 2019).
25. Hodgson, K.; Tansey, K.E.; Uher, R.; Dernovsek, M.Z.; Mors, O.; Hauser, J.; Souery, D.; Maier, W.; Henigsberg, N.; Rietschel, M.; et al. Exploring the role of drug-metabolising enzymes in antidepressant side effects. *Psychopharmacology (Berl).* **2015**, *14*, 2609–2617. doi: 10.1007/s00213-015-3898-x.
26. Bousman, C.; Byron, K.; Peh, L.H.; Smith, D.J.; Tan, C.H.; Ng, C.; Sarris, J.; Singh, A.; Schweitzer, I. Pharmacogenetic polymorphisms and response to escitalopram and venlafaxine over 8 weeks in major depression. *Hum. Psychopharmacol. Clin. Exp.* **2013**, *28*, 516–522.
27. Tsai, M.-H.; Lin, K.-M.; Hsiao, M.-C.; Shen, W.W.; Lu, M.-L.; Tang, H.-S.; Fang, C.-K.; Wu, C.-S.; Lu, S.-C.; Liu, S.C.; et al. Genetic polymorphisms of cytochrome P450 enzymes influence metabolism of the antidepressant escitalopram and treatment response. *Pharmacogenomics* **2010**, *11*, 537–546.
28. Brasch-Andersen, C.; Møller, M.U.; Christiansen, L.; Thinggaard, M.; Otto, M.; Brosen, K.; Sindrup, S.H. A candidate gene study of serotonergic pathway genes and pain relief during treatment with escitalopram in patients with neuropathic pain shows significant association to serotonin receptor2C (HTR2C). *Eur. J. Clin. Pharmacol.* **2011**, *67*, 1131–1137.
29. Bishop, J.R.; Najjar, F.; Rubin, L.H.; Guter, S.J.; Owley, T.; Mosconi, M.W.; Jacob, S.; Cook, E.H. Escitalopram pharmacogenetics: CYP2C19 relationships with dosing and clinical outcomes in autism spectrum disorder. *Pharmacogenet. Genomics.* **2015**, *11*, 548–554. doi: 10.1097/FPC.0000000000000173.
30. Kennisbank, Koninklijke Nederlandse Maatschappij ter bevordering der Pharmacie (KNMP), Version 3.0.5. Farmacogenetica. CLOMIPRAMINE CYP2D6 PM-IM-UM. Available online: https://kennisbank.knmp.nl/article/farmacogenetica/intro.html (accessed on 29 March 2019).
31. Vandel, P.; Haffen, E.; Nezelof, S.; Broly, F.; Kantelip, J.P.; Sechter, D. Clomipramine, fluoxetine and CYP2D6 metabolic capacity in depressed patients. *Hum. Psychopharmacol.* **2004**, *5*, 293–298.
32. Balant-Gorgia, A.E.; Balant, L.P.; Garrone, G. High blood concentrations of imipramine or clomipramine and therapeutic failure: A case report study using drug monitoring data. *Ther. Drug Monit.* **1989**, *11*, 415–420.
33. Stephan, P.L.; Jaquenoud Sirot, E.; Mueller, B.; Eap, C.B.; Baumann, P. Adverse drug reactions following nonresponse in a depressed patient with CYP2D6 deficiency and low CYP 3A4/5 activity. Pharmacopsychiatry. **2006**, *4*, 150–152.
34. Nielsen, K.K.; Brosen, K.; Gram, L.F.; Danish University Antidepressant Group. Steady-state plasma levels of clomipramine and its metabolites: Impact of the sparteine/debrisoquine oxidation polymorphism. *Eur. J. Clin. Pharmacol.* **1992**, *43*, 405–411.
35. Danish University Antidepressant Group. Clomipramine dose- effect study in patients with depression: clinical end points and pharmacokinetics. *Clin. Pharmacol. Ther.* **1999**, *2*, 152–165.
36. Bertilsson, L.; Dahl, M.L.; Sjögvit, F.; Aberg-Wistedt, A.; Humble, M.; Johansson, I.; Lundqvist, E.; Ingelman-Sundberg, M. Molecular basis for rational megaprescribing in ultrarapid hydroxylators of debrisoquine. *Lancet.* 1993, *8836*.
37. Baumann, P.; Broly, F.; Kosel, M.; Eap, C.B. Ultrarapid metabolism of clomipramine in a therapy-resistant depressive patient, as confirmed by CYP2 D6 genotyping. *Pharmacopsychiatry.* **1998**, *2*, 72.
38. Kennisbank, Koninklijke Nederlandse Maatschappij ter bevordering der Pharmacie (KNMP), Version 3.0.5. Farmacogenetica. NORTRIPTYLINE CYP2D6 IM-PM-UM. Available online: https://kennisbank.knmp.nl/article/farmacogenetica/intro.html. 2018 (accessed on 29 March 2019).

39. Chen, S.; Chou, W.-H.; Blouin, R.A.; Mao, Z.; Humphries, L.L.; Meek, Q.C.; Neill, J.R.; Martin, W.L.; Hays, L.R.; Wedlund, P.J. The cytochrome P450 2D6 (CYP2D6) enzyme polymorphism: Screening costs and influence on clinical outcomes in psychiatry. *Clin. Pharmacol. Ther.* **1996**, *60*, 522–534.
40. Roberts, R.L.; Mulder, R.T.; Joyce, P.R.; Luty, S.E.; Kennedy, M.A. No evidence of increased adverse drug reactions in cytochrome P450 CYP2D6 poor metabolizers treated with fluoxetine or nortriptyline. *Hum Psychopharmacol.* **2004**, *1*, 17–23.
41. Bertilsson, L.; Mellström, B.; Sjökvist, F.; Mårtenson, B.; Asberg, M. Slow hydroxylation of nortriptyline and concomitant poor debrisoquine hydroxylation: clinical implications. *Lancet.* **1981**, *8219*, 560–1.
42. Dahl, M.-L.; Bertilsson, L.; Nordin, C. Steady-state plasma levels of nortriptyline and its 10-hydroxy metabolite: relationship to theCYP2D6 genotype. *Psychopharmacology* **1996**, *123*, 315–319.
43. Dalén, P.; Dahl, M.-L.; Ruiz, M.L.B.; Nordin, J.; Bertilsson, L. 10-hydroxylation of nortriptyline in white persons with 0, 1, 2, 3, and 13 functional CYP2D6 genes. *Clin. Pharmacol. Ther.* **1998**, *63*, 444–452.
44. Kvist EE, Al-Shurbaji A, Dahl ML, Nordin C, Alván G, Ståhle L. Quantitative pharmacogenetics of nortriptyline: a novel approach. *Clin. Pharmacokinet.* **2001**, *40*, 869–877.
45. Murphy, G.M.; Pollock, B.G.; Kirshner, M.A.; Pascoe, N.; Cheuk, W.; Mulsant, B.H.; Reynolds, C.F.3rd. CYP2D6 genotyping with oligonucleotide microarrays and nortriptyline concentrations in geriatric depression. *Neuropsychopharmacol.* **2001**, *11*, 869–877.
46. Yue, Q.-Y.; Zhong, Z.-H.; Tybring, G.; Dalén, P.; Dahl, M.-L.; Bertilsson, L.; Sjöqvist, F. Pharmacokinetics of nortriptyline and its 10-hydroxy metabolite in Chinese subjects of different CYP2D6 genotypes. *Clin. Pharmacol. Ther.* **1998**, *64*, 384–390.
47. Lee, S.Y.; Sohn, K.M.; Ryu, J.Y.; Yoon, Y.R.; Shin, J.G.; Kim, J.W. Sequence-based CYP2D6 genotyping in the Korean population. *Ther. Drug Monit.* **2006**, *3*, 382–387.
48. Kennisbank, Koninklijke Nederlandse Maatschappij ter bevordering der Pharmacie (KNMP), Version 3.0.5. Farmacogenetica. SIMVASTATINE SLCO1B1 521TC-521CC. Available online: https://kennisbank.knmp.nl/article/farmacogenetica/intro.html (accessed on 29 March 2019).
49. SEARCH Collaborative Group; Link, E.; Parish, S.; Armitage, J.; Bowman, L.; Heath, S.; Matsuda, F.; Gut, I.; Lathrop, M.; Collins, R. SLCO1B1 variants and statin-induced myopathy -- a genomewide study. *N. Engl. J. Med.* **2008**, *8*, 789–799. doi: 10.1056/NEJMoa0801936.
50. Sortica, V.A.; Fiegenbaum, M.; Lima, L.O.; Van der Sand, C.R.; Van der Sand, L.C.; Ferreira, M.E.; Pires, R.C.; Hutz, M.H. SLCO1B1 gene variability influences lipid-lowering efficacy on simvastatin therapy in Southern Brazilians. *Clin. Chem. Lab. Med.* **2012**, *3*, 441–448. doi: 10.1515/cclm.2011.804.
51. Hu, M.; Mak, V.W.; Tomlinson, B. Intronic variants in SLCO1B1 related to statin-induced myopathy are associated with the low-density lipoprotein cholesterol response to statins in Chinese patients with hyperlipidaemia. *Pharmacogenetics Genom.* **2012**, *22*, 803–806.
52. Bailey, K.M.; Romaine, S.P.; Jackson, B.M.; Farrin, A.J.; Efthymiou, M.; Barth, J.H.; Copeland, J.; McCormack, T.; Whitehead, A.; Flather, M.D.; et al. Hepatic metabolism and transporter gene variants enhance response to rosuvastatin in patients with acute myocardial infarction: The GEOSTAT-1 Study. *Circ. Cardiovasc. Genet.* **2010**, *3*, 276–285. doi:10.1161/CIRCGENETICS.109.898502.
53. Kennisbank, Koninklijke Nederlandse Maatschappij ter bevordering der Pharmacie (KNMP), Version 3.0.5. Farmacogenetica. VENLAFAXINE CYP2D6 PM-IM-UM. Available online: https://kennisbank.knmp.nl/article/farmacogenetica/intro.html (accessed on 29 March 2019).
54. Shams, M.E.E.; Arneth, B.; Hiemke, C.; Dragicevic, A.; Müller, M.J.; Kaiser, R.; Lackner, K.; Härtter, S. CYP2D6 polymorphism and clinical effect of the antidepressant venlafaxine. *J. Clin. Pharm. Ther.* **2006**, *31*, 493–502.
55. McAlpine, D.E.; O'Kane, D.J.; Black, J.L.; Mrazek, D.A. Cytochrome P450 2D6 genotype variation and venlafaxine dosage. *Mayo Clin. Proc.* **2007**, *9*, 1065–1068.
56. van Nieuwerburgh, F.C.; Denys, D.A.; Westenberg, H.G.; Deforce, D.L. Response to serotonin reuptake inhibitors in OCD is not influenced by common CYP2D6 polymorphisms. *Int. J. Psychiatry Clin. Pract.* **2009**, *1*, 345–348.
57. Lobello, K.W.; Preskorn, S.H.; Guico-Pabia, C.J.; Jiang, Q.; Paul, J.; Nichols, A.I.; Patroneva, A.; Ninan, P.T. Cytochrome P450 2D6 phenotype predicts antidepressant efficacy of venlafaxine: A secondary analysis of 4 studies in major depressive disorder. *J. Clin. Psychiatry* **2010**, *11*, 1482–1487. doi:10.4088/JCP.08m04773blu.
58. Lessard, E.; A Yessine, M.; A Hamelin, B.; O'Hara, G.; Leblanc, J.; Turgeon, J. Influence of CYP2D6 activity on the disposition and cardiovascular toxicity of the antidepressant agent venlafaxine in humans. *Pharmacogenetics* **1999**, *9*, 435–443.
59. Fukuda, T.; Yamamoto, I.; Nishida, Y.; Zhou, Q.; Ohno, M.; Takada, K.; Azuma, J. Effect of the CYP2D6*10 genotype on venlafaxine pharmacokinetics in healthy adult volunteers. *Br. J. Clin. Pharmacol.* **1999**, *47*, 450–453.
60. Hermann, M.; Hendset, M.; Fosaas, K.; Hjerpset, M.; Refsum, H. Serum concentrations of venlafaxine and its metabolites O- desmethylvenlafaxine and N- desmethylvenlafaxine in heterozygous carriers of the CYP2D6*3, *4 or *5 allele. *Eur. J. Clin. Pharmacol.* **2018**, *5*, 483–487.

61. Kennisbank, Koninklijke Nederlandse Maatschappij ter bevordering der Pharmacie (KNMP), Version 3.0.5. Farmacogenetica. DOXEPINE CYP2D6 IM-PM-UM. Available online: https://kennisbank.knmp.nl/article/farmacogenetica/intro.html (accessed on 29 March 2019).
62. Kirchheiner, J.; Meineke, I.; Müller, G.; Roots, I.; Brockmöller, J. Contributions of CYP2D6, CYP2C9 and CYP2C19 to the biotransformation of Eand Z-doxepin in healthy volunteers. *Pharmacogenetics*. **2002**, *7*, 571–580.
63. Kirchheiner, J.; Henckel, H.-B.; Franke, L.; Meineke, I.; Tzvetkov, M.; Uebelhack, R.; Roots, I.; Brockmöller, J. Impact of the CYP2D6 ultra-rapid metabolizer genotype on doxepin pharmacokinetics and serotonin in platelets. *Pharmacogenetics Genom*. **2005**, *15*, 579–587.

© 2019 by the authors. Submitted for possible open access publication under the terms and conditions of the Creative Commons Attribution (CC BY) license (http://creativecommons.org/licenses/by/4.0/).
